# A Mutation Identified in Neonatal Microcephaly Destabilizes Zika Virus NS1 Assembly *in Vitro*

**DOI:** 10.1038/srep42580

**Published:** 2017-02-15

**Authors:** Deping Wang, Cheng Chen, Shengnan Liu, Han Zhou, Kailin Yang, Qi Zhao, Xiaoyun Ji, Chen Chen, Wei Xie, Zefang Wang, Li-Zhi Mi, Haitao Yang

**Affiliations:** 1 School of Life Sciences, Tianjin University, Tianjin 300072, People’s Republic of China; 2 Tianjin International Joint Academy of Biotechnology and Medicine, Tianjin 300457, People’s Republic of China; 3 Cleveland Clinic Lerner College of Medicine of Case Western Reserve University, Cleveland, OH 44195, USA; 4 Department of Molecular Biophysics and Biochemistry, Yale University, New Haven, CT 06520, USA

## Abstract

An unprecedented epidemic of Zika virus (ZIKV) infection had spread to South and Central America. ZIKV infection was recently confirmed by CDC (the Centers for Disease Control and Prevention) to cause neonatal microcephaly, which posed a significant public health emergency of international concern. No specific vaccines or drugs are currently available to fight ZIKV infection. ZIKV nonstructural protein 1 (NS1) plays an essential role in viral replication and immune evasion. We determined the crystal structure of ZIKV NS1_172–352_, which forms a head-to-head, symmetric dimer with a unique 14-stranded β-ladder conserved among flaviviruses. The assembly of the β-ladder dimer is concentration dependent. Strikingly, one pathogenic mutation T233A (NCBI accession no. KU527068), found in the brain tissue of infected fetus with neonatal microcephaly, is located at the dimer interface. Thr233, a unique residue found in ZIKV but not in other flaviviruses, organizes a central hydrogen bonding network at NS1 dimer interface. Mutation of Thr233 to Ala disrupts this elaborated interaction network, and destabilizes the NS1 dimeric assembly *in vitro*. In addition, our structural comparison of epitopes for protective antibody 22NS1, targeting West Nile Virus NS1, could potentially be valuable in understanding its anti-virus specificities and in the development of antibodies against ZIKV.

Zika virus (ZIKV) belongs to the arthropod-borne *Flavivirus genus*, which is composed of important human pathogens such as dengue (DENV), West Nile (WNV), and yellow fever (YFV)^1^. ZIKV was first isolated from the sentinel rhesus monkey in the Zika forest of Uganda in 1947 2. Sporadic case reports of ZIKV infection then occurred throughout Africa and Southeastern Asia, until the first epidemic outbreak in Yap Island in 2007 3. ZIKV infection is historically known to cause mild febrile flu-like symptoms in humans, characterized by fever, headache, arthralgia, myalgia, and maculopapular rash^4^. Leaving a series of widespread outbreaks in French Polynesia during 2013–2014 and in Brazil during 2015, the virus quickly spread out across Latin America and Caribbean countries^5^,^6^,^7^. Strikingly, more serious neurologic disorders, specifically microcephaly and Guillain-Barre syndrome^6^,^8^,^9^,^10^, had been associated with ZIKV epidemics in America and Oceania. As a consequence, World Health Organization (WHO) announced a Public Health Emergency of International Concern on February 1, 2016 11. Because of recent accumulation of evidences of ZIKV infection on pregnancy and fetal brain development, the CDC of the United States recently confirmed the causative link between ZIKV and microcephaly. No effective vaccines or therapeutics are currently available to prevent or manage ZIKV infection, placing an urgent call for accelerated research on ZIKV^12^.

The genome of ZIKV contains a 10.7-kb single-stranded, positive sense RNA. Similar to other flaviviruses, the genome of ZIKV encodes a single large polyprotein, which is proteolytically cleaved into 3 structural proteins (C, prM/M, and E), and 7 non-structural proteins (NS1, NS2A, NS2B, NS3, NS4A, NS4B, and NS5) by host and viral proteases^13^. The flavivirus NS1 is a ~48 kDa conserved non-structural protein with two conserved N-linked glycosylation sites and six intra-molecular disulfide bonds. NS1 synthesized in the ER lumen could form monomers, dimers, or hexamers after post-translational modification^14^,^15^,^16^. Intracellular NS1 located in the virus-induced organelles plays a key role in viral replication^17^,^18^. Moreover, NS1 could be secreted into the extracellular space either as a hexamer^16^, mimicking the structure of high-density lipoproteins^19^, or as a dimer associated with host plasma membrane^20^,^21^. Although not being a component of the virion, NS1 represents a marker for host immune recognition and evasion^22^.

Early structural studies of NS1s from DENV and WNV provided insights for NS1 assembly and its membrane association^23^,^24^. NS1 consists of the N-terminal β-roll, the middle wing, and the C-terminal β-ladder domains (Fig. 1A). Two NS1s are assembled into a symmetric, head-to-head dimer through domain swapping of the β-rolls and the extension of β-sheets from the β-ladders (Fig. 1B and C)^23^. The β-rolls are required for lipid packing in the hexamer and cell attachment in the dimer, while the β-ladders and wings are exposed for immune recognition (Fig. 1B and C)^23^,^24^.

Recent progress in the structural studies of ZIKV NS1 revealed that the β-rolls and two aromatic loops, one from each Wing domain, in together from the hydrophobic anchor for membrane association^25^. In addition, the polar surface on the β-ladder side varies substantially in their electrostatic properties, especially at the central region of the β-ladder dimer, among flaviviruses^26^.

To understand the function of ZIKV NS1, we solved the crystal structure of ZIKV NS1_172–352_ from ZIKV strain Suriname (KU312312). The structure is composed of a head-to-head dimer with a 14-stranded β-ladder conserved among flaviviruses. A detailed analysis of the variation and mutations in the NS1 dimer interface provides insights into NS1 assembly and molecular pathogenesis. Structural comparison of the epitopes for the protective antibody 22NS1, targeting West Nile Virus NS1, could potentially be valuable in the development of antibodies against ZIKV.

## Results

### The overall structure of ZIKV NS1 β-ladder domain

The crystal of ZIKV NS1 β-ladder domain (NS1_172–352_) diffracted to a 2.8 Å resolution in Fourier space. Two β-ladder protomers were found in each asymmetric unit. These two protomers form a symmetric, head-to-head dimer through an extended, 14-stranded β-sheet seen in the dimeric assembly of DENV and WNV NS1 β-ladder structures (Fig. 2A)^23^,^24^. In a manner reminiscent of railroad ties, these 14 β-strands laid the structural foundation for the NS1 β-ladder dimer. Cross the “railroad ties”, spaghetti loops and a short α-helix link these β-strands together, and form the exposed surface for immune recognition^24^. At the two distal ends, each protomer contains 2 additional β-strands capping the β-ladder.

### The dimeric assembly of ZIKV NS1

While the NS1 β-ladder domains of flaviviruses share a well conserved overall structure, they did show noticed flexibility at the dimer interface and at the two distal ends of the dimer (Fig. 2B and C). The C_α_ RMSD between ZIKV and WNV NS1_176–351_ monomers ranges from 0.82 Å to 0.87 Å, but the C_α_ RMSD of the dimer raises to 0.91 Å. Similar results are given in comparison of ZIKV and DENV NS1 structures. In agreement with these calculations, the association angle between two monomers in the dimer, as defined by the dihedral angle of the two β-ladders protomers, varies slightly in different structures (Fig. 2C). This flexibility may arise from crystal packing at different crystallization conditions, or rather simply reflects the difference of these structures at the dimer interface (Fig. 2D).

**Figure 1:**
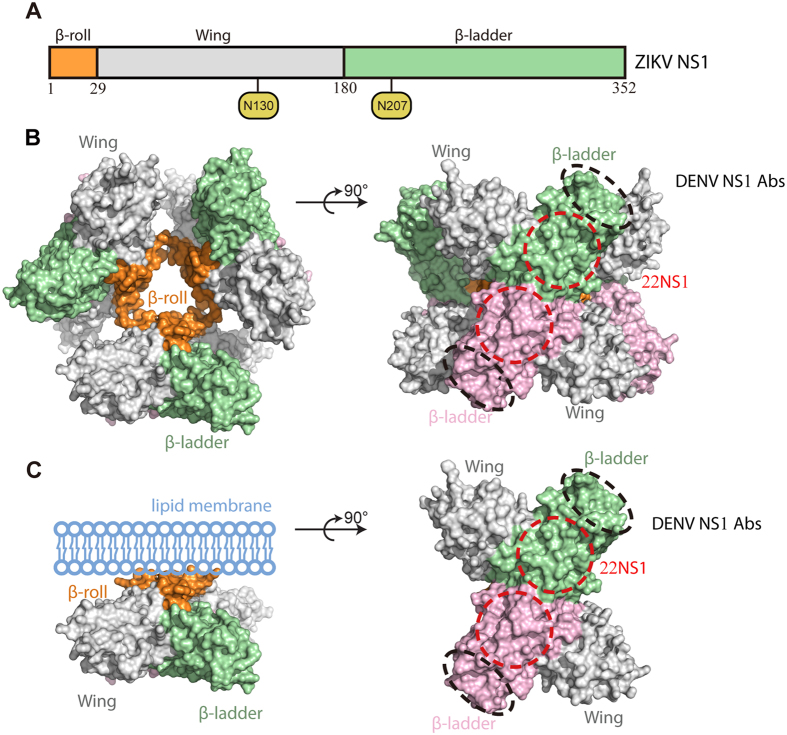
Schematic diagrams of flavivirus NS1 structure and assemblies. (**A**) The primary structure of ZIKV NS1. The glycosylation sites are shown in yellow circles. (**B**) The hexameric assembly of flavivirus NS1. The surface model is generated from the crystallographic symmetry of DENV NS1 (pdb ID 4O6B)^23^. The β-rolls are orange; the Wings are grey. The dimeric β-ladders are shown with one monomer in green and the other in pink. The epitopes for pathogenic antibodies against DENV are circled with black dash lines^43^. The epitopes for protective antibody 22NS1 against WNV are circled with red dash lines^24^. (**C**) The dimeric assembly of flavivirus NS1. The surface model of NS1 dimer is generated from the structure of DENV NS1 (pdb ID 4O6B)^23^. The lipid membrane is light blue.

**Figure 2:**
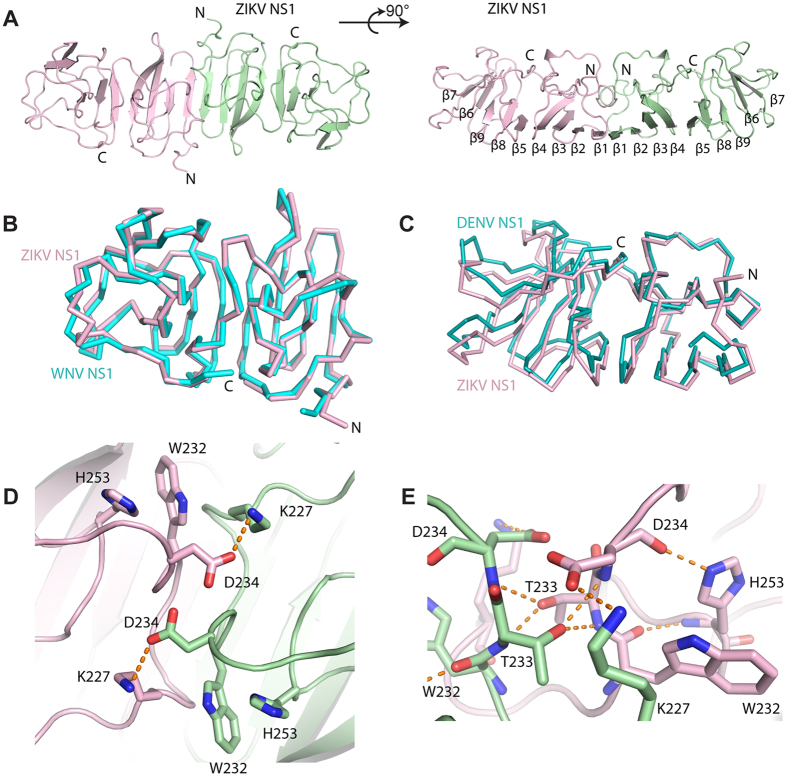
The structure and dimeric assembly of ZIKV NS1. (A) The crystal structure of ZIKV NS1 β-ladder in two different orientations. The structure is shown in cartoon with one monomer in pink and the other in green. (**B**) Comparison of ZIKV and WNV NS1 dimer structures. WNV NS1 dimer (pdb ID 4OII)^24^ and ZIKV NS1 dimer are superimposed on their β-ladders. For clarity, only one monomer from each dimer is shown in ribbon representation at the same orientation as the figure above. (**C**) Comparison of the dimeric assemblies of flavivirus NS1 β-ladders. The dimeric structures of ZIKV NS1 and DENV NS1 (pdb ID 4OIG)^24^ are superimposed on one monomer from each dimer. For clarity, the superimposed monomers are not shown. The other monomers are represented in ribbon at the same orientation as the figure above. (**D**) The details of ZIKV NS1 β-ladder domain dimer interface. The variable residues at the dimer interface are represented in sticks. Hydrogen bonds are shown in orange dash lines. (**E**) Two T233s at NS1 dimer interface organizing a hydrogen bond network.

**Figure 3:**
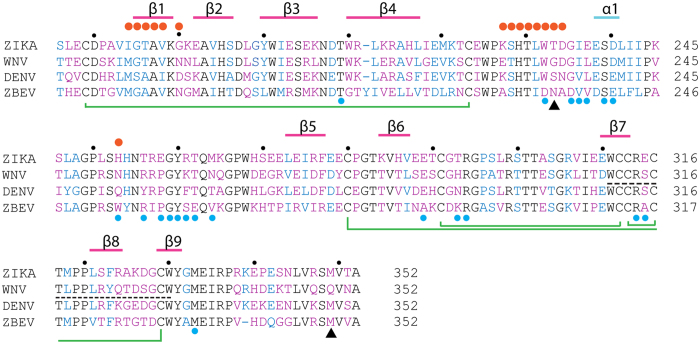
The sequence alignment of flavivirus NS1 β-ladders. An α-helix is marked with a cyan line, β-strands are marked with magenta lines. Disulfide bonds are connected with green lines. In the alignment, conserved residues are black; similar residues are blue; variable residues are purple. Black dots, the 10-digit residues; orange dots, residues at the dimer interface; blue dots, epitope residues recognized by 22NS1^24^; black dash line, epitope for pathogenic antibodies^43^; black triangles, the residues mutated in Natal_RGN strain^6^.

The dimer interface buries a total surface area of 1628 Å^2^. Except K227, D234, W232, and H253, other residues are conserved among flaviviruses, indicating the physiological relevance of the assembly (Fig. 3). In ZIKV NS1, K227 forms a salt bridge with D234, while H253 interacts with W232 through a π-π stacking interaction at physiological pH (Fig. 2D). Substitution of Lys227 to Glu, and His253 to Asn, in WNV disrupt these interactions, and may reduce the interaction strength of the dimeric assembly (Fig. 3). The residues at the flexible C-terminal ends are variable in sequence, consistent with their roles as frequently-mapped epitopes in WNV immune reactions (Figs 1B,C,2B and 3)^28^.

Remarkably, T233A, one of the mutations found in the Natal_RGN strain (KU527068), which was isolated from fetal brain tissue with severe microcephaly^6^, is located at the NS1 dimer interface. The hydroxyl group of T233 is central in organizing a hydrogen-bonding network across the dimer interface (Fig. 2E). T233 directly forms hydrogen bonds with the backbone amine groups of D234 and T233, reside on the flexible β4-α1 loop of the other monomer. As such, the side chains of D234 and W232 are positioned to form a salt bridge with K227, and a π-π stacking interaction with H253, respectively. It is worthwhile to note that our structure is very similar to other published ZIKV NS1 structures with the root-mean-square deviations ranging from 0.4–0.6 Å (for Cα; PDB IDs: 5GS6, 5IY3 and 5K6K) (Supplementary Figure 1A). In particular, it has been shown that T233 also plays a pivotal role in organizing a hydrogen-bonding network across the dimer interface in other solved ZIKV NS1 structures (Supplementary Figure 1B). These structural data suggests that mutation of Thr233 to Ala would disrupt this elaborated network, and could potentially impair the stability of the dimer.

**Figure 4:**
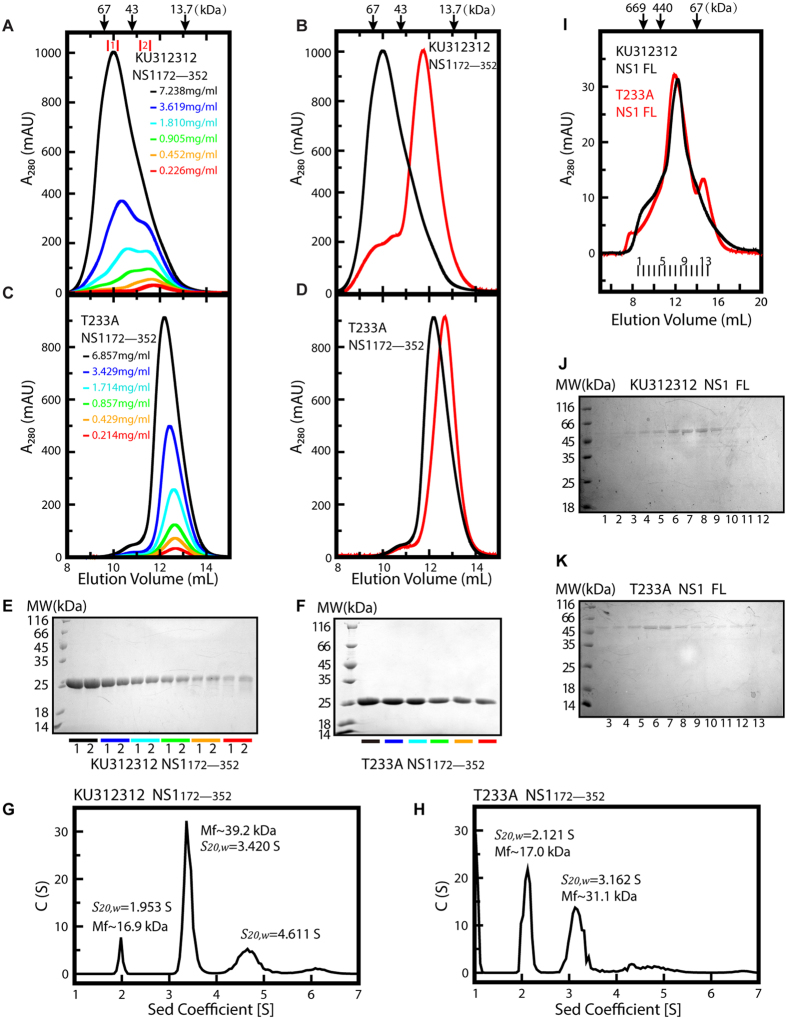
T233A mutation destabilizes the dimeric assembly of ZIKV NS1. (**A–D**) The overlaid gel-filtration chromatography profiles at different ZIKV NS1 protein concentrations. (**A,C**)The purified, refolded ZIKV NS1 from ZIKV strain Suriname (KU312312) (**A**) and T233A mutant (**C**) proteins were subjected to 2-fold serial dilution, and then each diluted sample was analyzed by gel-filtration chromatography using a Superdex 75 10/300 GL column. (**B,D**) The gel-filtration chromatography profiles of the Suriname NS1 (**B**) and T233A mutant (**D**) NS1 at the highest and the lowest protein concentrations are scaled to the same relative peak height for comparison. (**E,F**) Coomassie-stained reducing SDS-PAGE of fractionated Suriname NS1 (**E**) and T233A mutant (**F**) protein samples from each gel-filtration chromatography. (**G,H**) Analytical ultracentrifugation analysis of the Suriname (**G**) and T233A mutant (**H**) NS1 β-ladder domains. The experiments were carried out at protein concentration of 2.95 mg/mL under the rotational speed of 53,000 rpm. (**I,J,K**) The size-exclusion chromatography profiles of the Suriname and T233A mutant full-length NS1 at the concentration of 0.47 mg/mL and 0.43 mg/mL, respectively. The eluates of the parent (**J**) and T233A mutant (**K**) NS1 were then subjected to Coomassie-stained SDS-PAGE analysis.

Consistent with our structural analysis, we found the Suriname NS1 β-ladder is assembled in a concentration dependent manner. Lowering the concentration of purified Suriname NS1 β-ladder by 2-fold serial dilution gradually shifted the equilibrium between monomer and dimer from a dimer-dominant profile to a monomer-dominant profile in size-exclusion chromatography (Fig. 4A,B and E). In contrast, even at 6.9 mg/ml, the highest concentration tested in our experiments, the size-exclusion chromatography profile of purified T233A mutant showed only a single, symmetric peak at a smaller stokes radius (1.93 nm) than that of the Suriname NS1 monomer (2.06 nm) (Fig. 4B,C,D,F,G and H). Moreover, by lowering the concentration of purified T233A mutant by 2-fold serial dilution, we noticed the apparent molecular weight of the protein slightly but reproducibly decreased in a concentration dependent manner, indicating fast dissociation kinetics in the dimeric assembly of T233A mutant (Fig. 4C,D and H).

In agreement with the size exclusion chromatography results, the analytical ultracentrifugation experiments also indicated that T233A mutation destabilizes the dimeric assembly of the NS1 β-ladder domain (Fig. 4G and H). At the concentration of 2.95 mg/ml, the Suriname NS1 β-ladder exhibited three oligomeric states: monomer, dimer, and a higher order oligomer, in analytical ultracentrifugation with sedimentation coefficient (S_20, w_) of 1.953 S, 3.420 S, and 4.611 S, respectively (Fig. 4G). The monomeric proteins only account for 7.8% of the total population of the three oligomeric states. In contrast, the analytical ultracentrifugation profile of the T233A mutant at the same concentration showed two majors peaks, corresponding to the monomer and the dimer with sedimentation coefficient of 2.121 S, and 3.162 S, respectively (Fig. 4H). Over 49% of T233A mutant are in the monomeric state. This percentile is much higher than that of the parent Suriname NS1 β-ladder domain, suggesting that T233A mutation interferes the self-association of ZIKV NS1 β-ladder domain.

T233A mutation had a moderate impact on the self-assembly of the full-length NS1 expressed from insect cells. At the concentration of 0.47 mg/ml, the parent NS1 was eluted as a single peak, at an apparent molecular weight of 130 kDa, in size-exclusion chromatography (Fig. 4I and J). In comparison, the size-exclusion chromatography profile of the T233A mutant was split into two peaks, a major peak at an apparent molecular weight of 150 kDa, and a minor peak at an apparent molecular weight of 40 kDa, indicating that a small fraction of T233A NS1 remained in monomeric state (Fig. 4I and K).

### The epitope for WNV protective antibody 22NS1

**Figure 5:**
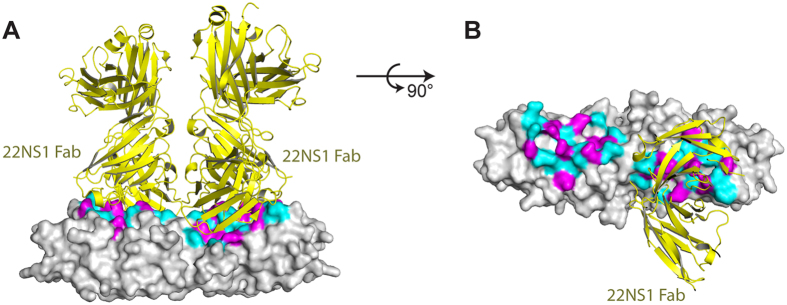
The epitope for WNV protective antibody 22NS1. (**A,B**) The structures of 22NS1-bound WNV NS1 (pdb ID 4OII)^24^ and ZIKV NS1 are superimposed on their β-ladder dimers. ZIKV NS1 is represented in grey surface, and the 22NS1 is represented in yellow cartoon. The variable residues on the epitope are magenta, and the identical residues are cyan. For clarity, one 22NS1 is omitted in panel b.

The epidemic emergency of ZIKV necessitates the expedited development of therapeutics in diagnosis and management of the virus infection. It is an intriguing question whether the existing protective antibodies targeting other flaviviruses could be reengineered or even directly utilized to neutralize ZIKV. In this regard, we compared the structures of ZIKV NS1 and Fab-bound WNV NS1. The Fab was derived from the protective antibody 22NS1, which had been evaluated in animal models^22^. Simultaneous administration of 22NS1 and WNV could protect the mice from lethal infection^22^. The epitope for 22NS1 was located on the spaghetti loop side of each monomer. Out of 21 residues on the epitope, 13 were identical to the corresponding residues in ZIKV. The rest variable residues on the epitope, and corresponding recognition contacts on the antibody, were shown in Figs 3 and 5, and were listed in Table 1. This structural information provides a clue for future rational design and engineering of antibodies against ZIKV NS1.

## Disscussion

Our structural study of ZIKV NS1 provided a framework for understanding the assembly and immune recognition of this critical viral protein, which serves as a marker in serum or on the cell surface of infected host for immune recognition^29^. In contrast to the newly-synthesized, monomeric form, extracellular NS1s assemble into dimers or lipoprotein-like hexamers^14^. In our structure, the ZIKV NS1 β-ladder domain by itself forms a head-to-head dimer, consistent with reported DENV and WNV NS1 structures as well as the newly published ZIKV NS1 structures^23^. The overall structure of NS1 β-ladder is well conserved among flaviviruses. However, notable flexibility was seen in the dimerization and on the C-terminal loop. Strikingly, T233A, one of the unique mutations found in the Natal_RGN strain, is located at the dimer interface. Our structure explains that T233A mutation could disrupt an elaborated hydrogen-bonding network across the dimer interface. Consistent with this explanation, our biochemical data demonstrated that the assembly of ZIKV NS1 β-ladder dimer is concentration dependent. T233A mutation interferes the self-association of NS1 β-ladder domain as well as the full-length NS1. This interference is probably, at least in part, caused by the acceleration of the dissociation kinetics of NS1 dimer, as evidenced by size-exclusion chromatography and analytical ultracentrifugation experiments. It is equally possible, though, that T233A mutation affects the overall conformation of NS1, as the stokes radius of the monomeric T233A mutant β-ladder domain is smaller than that of its parent β-ladder monomer. In the future, it will be worth to investigate the functional and pathogenic impacts of destabilized ZIKV NS1 assembly and/or possibly conformational variation induced by T233A mutation on the secretion of NS1 as the marker for infection, and on the immune evasion of ZIKV.

Indeed, high serum level of NS1 in the case of DENV infection correlates with more severe clinical courses, including dengue hemorrhagic fever and dengue shock syndrome^30^,^31^. Although the exact pathogenesis remains unclear, extracellular NS1 is proposed to promote immune complex formation, causing endothelial leakage through binding to endothelial cell wall and triggering antibody-mediated damage^32^,^33^. This will decrease complement recognition of infected cells^34^, modulate cellular metabolism^35^, and directly facilitate viral infection^36^.

A unique feature of ZIKV is to cross the placenta and infect neural stem cells in the developing fetal brain, which distinguishes it from other flaviviruses^11^,^37^,^38^. Although ZIKV has been circulating in Africa and Southeastern Asia for more than half a century, the recent emergence of microcephaly and other congenital neurologic defects led to the hypothesis that it might have increased neurotropism and enhanced capacity for transmission/proliferation during viral evolution^11^. In a recent comparative genomic analysis of ZIKV, almost 50% of the unique nucleotide mutations found in the Natal_RGN strain reside in the NS1 protein. This observation raises an interesting point of tissue-specific mutation for viral tropism in ZIKV infection, which has been reported in the hepatitis C virus infection^39^,^40^. Besides the T233A mutation at NS1 dimer interface, two other mutations found in the Natal_RGN strain, K146E and M349V, are located at the NS1 wing domain and at the flexible C-terminal loop of the β-ladder, respectively. The pathological and immunological significance of observed mutations in NS1 require further investigation.

The lack of therapeutics against ZIKV challenge attracts global concerns during the outbreak of ZIKV transmission. The NS1 of DENV and WNV had been explored in the vaccination or in the generation of protective antibodies at preclinical settings^22^,^41^. The evaluation results, however, are promiscuous. Some antibodies are pathogenic^43^, while a few of others are protective^22^,^41^. The epitope for some pathogenic antibodies against DENV, had been mapped to the C-terminal end on each NS1 monomer^43^, while the epitope for one protective antibody against WNV, 22NS1, had been mapped to the central region on each NS1 monomer^24^ (Figs 1B and 3). The underlying mechanisms for their differentiating pathogenic or therapeutic effects need to be clarified. It had been suggested that the linear epitope on DENV NS1 could elicit autoimmune reactions as it shares high sequence similarity with host autoantigens^43^. In contrast, 22NS1 recognizes a steric epitope on WNV NS1, and triggers the clearance of infected host cells through Fc gamma receptor pathway^22^,^24^,^41^. Our structural comparison of ZIKV NS1 with protective antibody bound WNV NS1 provided a starting point for rational design and engineering of antibodies against ZIKV NS1, which could be potentially valuable in the diagnosis or protection of ZIKV.

## Methods

### Cloning and expression of ZIKV NS1 β-ladder domain

The ZIKV NS1 β-ladder domain(residues 172 to 352) (NCBI accession no. KU312312) was cloned into the NheI and NotI restriction sites of pET21d for expression in the *Escherichia coli* BL21(DE3) codon plus. Cells were grown in LB medium, and then induced with 1 mM isopropyl-β-D-thiogalactopyranoside for 4 hours at 37 °C. The cells were harvested by centrifugation at 3,470 g for 20 minutes.

### Purification and crystallization of ZIKV NS1 β-ladder domain

The pellet of *E. coli* cells transformed with plasmids encoding ZIKV NS1_172–352_ was resuspended in PE buffer (20 mM NaH_2_PO_4_, 20 mM K_2_HPO_4_, 1 mM EDTA, pH 7.2) and sonicated three times on ice for 10 minutes each at 35% power. The lysate were cleared by centrifugation at 17,418 g for 10 minutes. The pellet was collected and washed successively with 2 M urea, Triton X-100/EDTA (0.5% Triton X-100, 10 mM EDTA), PE buffer, and TE buffer (20 mM Tris-HCl, 1 mM EDTA, pH 8.0). The washed pellet was solubilized in a buffer containing 7 M guanidinium hydrochloride and 10 mM β-mercaptoethanol for 2 hours at 37 °C. The solution was diluted by 3.5 folds with 50 mM sodium acetate at pH 5.2. Then, 100 mg protein solution was slowly titrated into 1 L refolding buffer (400 mM L-arginine, 100 mM Tris-base pH 8.3, 2 mM EDTA, 0.5 mM oxidized glutathione, 5 mM reduced glutathione, and 0.2 mM phenylmethanesulfonyl fluoride) at a flow rate of 0.02 mL/min. After titration, the solution was cleared by centrifugation at 17,418 g for 10 min at 4 °C. The proteins were then applied to a 5 mL HiTrap Q column (GE) pre-equilibrated with buffer A (50 mM Tris-HCl, pH 8.0), and were fractionated by using a linear NaCl concentration gradient. The fractions containing ZIKV NS1 were pooled and subjected to two successive gel-filtration chromatography purification steps using a Superdex 75 10/300 GL column (GE) equilibrated in 20 mM Hepes, pH 7.4, and 150 mM NaCl.

ZIKV NS1_172–352_ was crystallized at 18 °C by hanging-drop vapor diffusion in 0.1 M MES monohydrate, pH 6.0, 20% (w/v) Polyethylene glycol monomethyl ether 2,000, and 20% (v/v) 2-Propanol. The crystallization conditions were further optimized. The crystals were cryo-protected in 0.1 M MES monohydrate pH 6.0, 14% (w/v) Polyethylene glycol monomethyl ether 2,000, 18% (v/v) 2-Propanol, and 25% (w/v) glycerol.

### Cloning, expression and purification of the full-length ZIKV NS1s

The full-length ZIKV NS1 (1–352aa) with an Op64 signal peptide was subcloned into pFASTBac HTA vector from Invitrogen^44^. The site-directed mutagenesis of T233A mutation was conducted with Mut ExpressTM II Fast Mutagenesis Kit (Vazyme). The recombinant bacmids were genereated by transforming 25 μL of DH10 Bac cells (Invitrogen) with 1 μL plasmids encoding the parent or T233A mutant ZIKV NS1. Transfection and virus amplification were performed according to the manual from the manufacture (Invitrogen). Soluble NS1 proteins were produced by infecting suspension cultures of sf9 cells (Invitrogen) for 72 hours. The supernatant was collected and loaded on a Ni Sepharose (GE) affinity column equilibrated with buffer A (50 mM Tris pH8.5, 50 mM (NH4)_2_SO_4_, 10% glycerol). Bound proteins were eluted from the column using buffer A supplemented with 200 mM imidazole. The fraction containing NS1 proteins was then loaded onto a 5 mL HiTrap Q column (GE) pre-equilibrated with buffer B (50 mM Tris-HCl, pH 8.0) and eluted using a linear NaCl concentration gradient. The protein of interest was concentrated and subjected to a gel-filtration chromatography purification using a Superdex 200 column (GE) equilibrated in running buffer C (20 mM Hepes, pH 7.4, 150 mM NaCl). The eluates from the gel-filtration chromatography were further analyzed by Coomassie-stained SDS–PAGE.

### Analytical ultracentrifugation analysis of NS1 β-ladder domain self-association

Sedimentation velocity experiments were performed in a ProteomeLab XL-I analytical ultracentrifuge (Beckman Coulter, Brea, CA), equipped with AN-60Ti rotor (4-holes) and conventional double-sector aluminum centerpieces with 12 mm optical path length^45^. 400 μL of sample and 400 μL of buffer (20 mM Hepes, 150 mM NaCl, pH 7.4) were loaded in each experiment. The parent and T233A mutant NS1 β-ladder, at the concentration of 2.95 mg/mL, were analyzed in a buffer containing 20 mM Hepes pH 7.4, and 150 mM NaCl. Before the experiments, the rotor was pre-equilibrated for approximately 1 h at 20 °C in the centrifuge. All experiments were carried out at 20 °C under the rotational speed of 53,000 rpm. Optical interference was used for sample detection. Scans were collected at every 3 minutes. The data was fitted with a continuous sedimentation coefficient distribution model, covering a range of 0.5–10 S, using SEDFIT software (https://sedfitsedphat.nibib.nih.gov/software)^45^. Biophysical parameters used in data fitting were follows: buffer density ρ = 1.0000 g/cm^3^, buffer viscosity η = 0.01002, and proteins’ partial specific volume V-bar = 0.73000 cm^3^/g^45^.

### X-ray diffraction data collection, structure determination and refinement

Diffraction data were collected at 100 K at Shanghai Synchrotron Radiation Facility (SSRF) beamline BL19U1. The Diffraction data were collected at a wavelength of 0.97853 Å and processed with HKL2000 46. The structure was solved with PHENIX^47^ by molecular replacement using the structure of PDB ID 4O6C^24^ as the search model. Multiple rounds of model building in COOT and refinement in PHENIX were performed. The data collection and refinement statistics are summarized in Supplementary Table 1.

### Structural Analysis and Illustrations

COOT^48^ and PYMOL (The PyMOL Molecular Graphics System, Version 1.8 Schrödinger, LLC) were used for the structural analysis and illustration.

## Additional Information

**How to cite this article**: Wang, D. *et al*. A Mutation Identified in Neonatal Microcephaly Destabilizes Zika Virus NS1 Assembly *in Vitro. Sci. Rep.* **7**, 42580; doi: 10.1038/srep42580 (2017).

**Publisher's note:** Springer Nature remains neutral with regard to jurisdictional claims in published maps and institutional affiliations.

## Supplementary Material

Supplementary Information

## Figures and Tables

**Table 1:** The variable residues on NS1 epitope and their recognition contacts of the antibody 22NS1.

Variable residues on NS1 epitope: WNV (ZIKV)^a^	Heavy chain contacts	Light chain contacts
L237 (E237)	D50, W47	T94, R96
N253 (H253)	—	W92
R256 (T256)	Y98	W92, F91, Y32
P258 (E258)	—	Y32
K261 (R261)	Y98, D95	—
N264 (M264)	N63	—
H293 (T293)	Y27, V2, Y32	—
S315 (E315)	D96, R99	Y49

^a^The variable residues on the WNV NS1 epitope recognized by the protective antibody 22NS1 are listed outside of parentheses, while the corresponding residues of ZIKV NS1 are listed in parentheses.
