# Supplementary material for: A Mutation Identified in Neonatal Microcephaly Destabilizes Zika Virus NS1 Assembly in Vitro
Source: Sci Rep. 2017 Feb 15;7:42580. doi: 10.1038/srep42580 (PMC5309781; doi:10.1038/srep42580)
Supplement: Supplementary Information [file srep42580-s1.pdf]

A Mutation identified in Neonatal Microcephaly Destabilizes Zika Virus NS1 Assembly *in vitro*

Deping Wang,[1,+] Cheng Chen,[1,2,+] Shengnan Liu,[1,+] Han Zhou,[1,+] Kailin Yang,[3] Qi Zhao,[4] Xiaoyun Ji,[4] Chen Chen,[1] Wei Xie,[1] Zefang Wang,[1,2] Li-Zhi Mi[1,*], Haitao Yang[1,2,*]

[1]School of Life Sciences, Tianjin University, Tianjin 300072, People's Republic of China

[2]Tianjin International Joint Academy of Biotechnology and Medicine, Tianjin 300457, People's Republic of China

[3]Cleveland Clinic Lerner College of Medicine of Case Western Reserve University, Cleveland, OH 44195, USA

[4]Department of Molecular Biophysics and Biochemistry, Yale University, New Haven, CT 06520, USA

[+]These authors contributed equally to this work.

*Correspondence: yanght@tju.edu.cn, lizhi.mi@tju.edu.cn

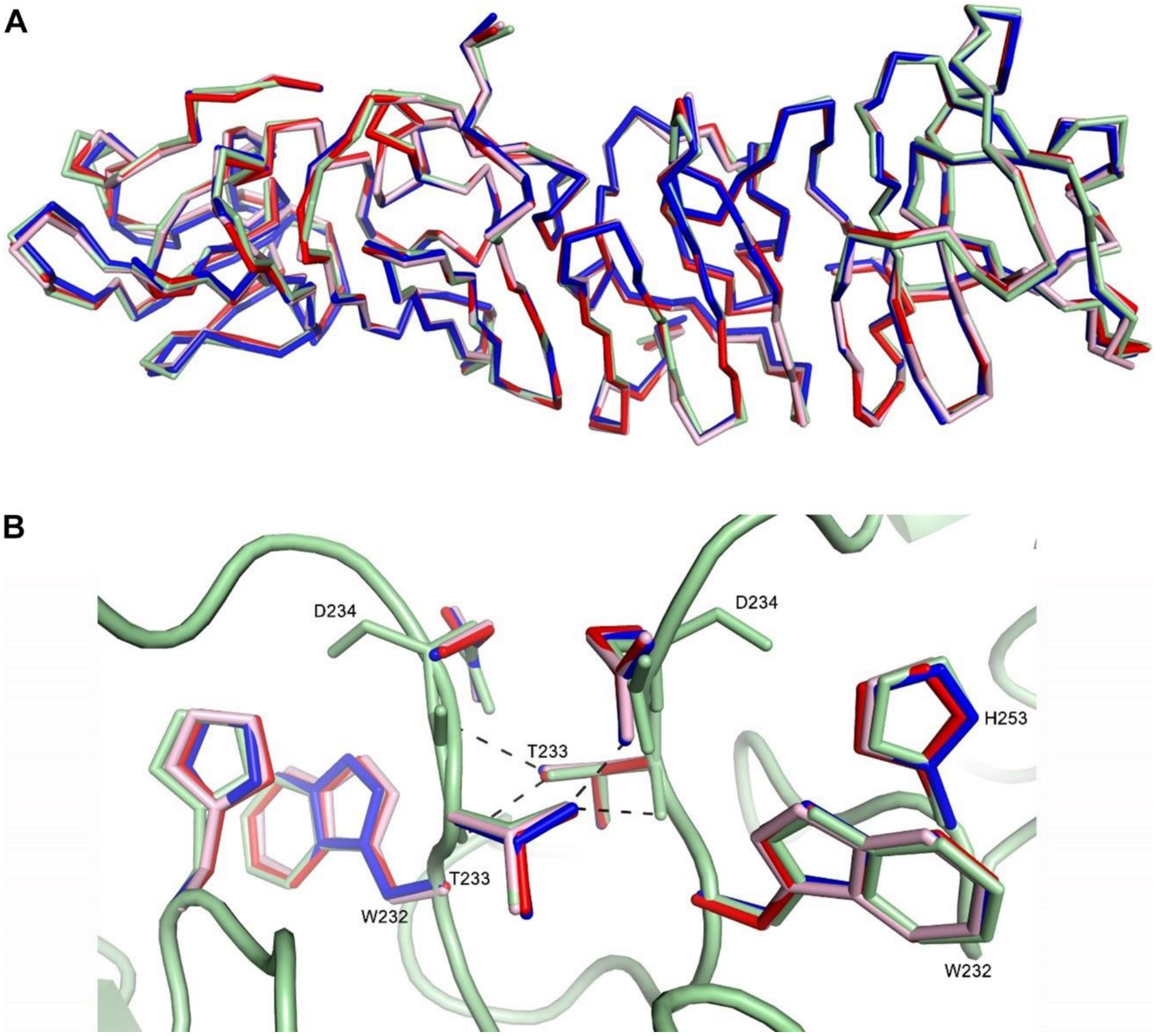

Supplementary Figure 1. Comparison of the C-terminal structures from ZIKV NS1.

(A) The overlay of the main chain of four ZIKV NS1 C-terminal structures. The Suriname strain in green; PDB code: 5GS6 in red, PDB code: 5IY3 in blue, PDB code: 5K6K in pink [1,2,3].

(B) T233 plays a pivotal role in organizing a hydrogen-bonding network across the dimer interface among all ZIKV NS1 structures. The hydrogen bonds are shown in dash line.

Supplementary Table 1. Data collection and refinement statistics

	$NS1_{172–352}$
**Data Collection**	
X-ray Source	SSRF beamline BL19U1
Wavelength (Å)	0.97853 Å
Space group	$C222_1$
Unit cell a,b,c,α,β,γ (Å)	63.9, 100.8, 110.7, 90.0, 90.0, 90.0
Resolution range (Å)	50.0-2.80 (2.88-2.80) [a]
Unique reflections	8937 (705) [a]
Completeness (%)	99.4 (96.4) [a]
Redundancy	12.0 (9.6) [a]
$I/\sigma I$	15.9 (2.8) [a]
R_{merge} [b]	0.134 (0.653) [a]
**Refinement**	
Resolution range (Å)	37.27-2.82
No. of reflections (working/test)	7756/790
R_{work}/R_{free} [c]	0.210/0.259 (0.221/0.322)
Number of atoms	
Protein/water	2810/4
B-factors	
Protein	54.7
Water	20.3
r.m.s. deviations	
Bond lengths (Å)	0.004
Bond angles (°)	0.69
Ramachandran Plot [d]	
(% favored/allowed/outliers)	95/5/0
Rotamer Outliers (%) [d]	0.64
Clashscore [d]	6.88

[a] Values in parentheses are the statistics for the highest resolution shell.

[b] $R_{merge} = \Sigma_{hkl}\Sigma_j|\mathbf{I}_{hkl} - \mathbf{I}_{hkl}(J)|/\Sigma_{hkl}\Sigma_j|\mathbf{I}_{hkl}(J)|$. $\mathbf{I}_{hkl}(J)$ and $\mathbf{I}_{hkl}$ represent the jth and mean intensity of reflection hkl.

[c] $R_{work} = \Sigma_{hkl}||\mathbf{F}_{obs}|-|\mathbf{F}_{calc}||/|\mathbf{F}_{obs}|$. $\mathbf{F}_{obs}$ and $\mathbf{F}_{calc}$ represent the observed and calculated structure factors, respectively. R_{free} is the R factor calculated with 10 % of unique reflections as the test set.

[d] The values are reported by PHENIX.

References

1 Xu X. *et al.* Contribution of intertwined loop to membrane association revealed by Zika virus full length NS1 structure. *EMBO J* **35,** 2170-2178 (2016), doi: 10.15252/embj.201695290.

2 Brown W.C. *et al*. Extended surface for membrane association in Zika virus NS1 structure. *Nat. Struct Mol Biol* **23,** 865-867 (2016), doi: 10.1038/nsmb.3268.

3 Song, H., Qi, J., Haywood, J., Shi, Y. & Gao, G. F. Zika virus NS1 structure reveals diversity of electrostatic surfaces among flaviviruses. *Nat Struct Mol Biol* **23**, 456-458 (2016), doi: 10.1038/nsmb.3213.
